# Contraceptive Access and Use Among Undergraduate and Graduate Students During COVID-19: Online Survey Study

**DOI:** 10.2196/38491

**Published:** 2023-03-14

**Authors:** Emily Chen, Adam Hollowell, Tracy Truong, Keisha Bentley-Edwards, Evan Myers, Alaattin Erkanli, Lauren Holt, Jonas J Swartz

**Affiliations:** 1 Duke University School of Medicine Durham, NC United States; 2 Samuel Dubois Cook Center on Social Equity Duke University Durham, NC United States; 3 Department of Biostatistics & Bioinformatics Duke University Durham, NC United States; 4 Department of General Internal Medicine Duke University Medical Center Durham, NC United States; 5 Department of Obstetrics & Gynecology Duke University Medical Center Durham, NC United States; 6 School of Nursing Duke University School of Nursing Durham, NC United States

**Keywords:** COVID-19, contraception, college, disparities, LARC, sexual health, social media, health promotion, telehealth, health messaging, health resource, health disparity, risk factor, healthcare access

## Abstract

**Background:**

The COVID-19 pandemic led to widespread college campus closures in the months of March to June 2020, endangering students’ access to on-campus health resources, including reproductive health services.

**Objective:**

To assess contraceptive access and use among undergraduate and graduate students in North Carolina during the COVID-19 pandemic.

**Methods:**

We conducted a cross-sectional web-based survey of undergraduate and graduate students enrolled at degree-granting institutions in North Carolina. Participants were recruited using targeted Instagram advertisements. The survey queried several aspects of participants’ sexual behavior, including sex drive, level of sexual experience, number of sexual partners, digital sexual experience, dating patterns, and types of contraception used. Participants were asked to compare many of these behaviors before and after the pandemic. The survey also assessed several sociodemographic factors that we hypothesized would be associated with contraceptive use based on prior data, including educational background, sexual orientation and gender minority status (ie, lesbian, gay, bisexual, transgender, queer), health insurance status, race, ethnicity, degree of sensation seeking, religiosity, and desire to become pregnant.

**Results:**

Over 10 days, 2035 Instagram users began our survey, of whom 1002 met eligibility criteria. Of these 1002 eligible participants, 934 completed the survey, for a 93% completion rate. Our respondents were mostly female (665/934, 71%), cisgender (877/934, 94%), heterosexual (592/934, 64%), white (695/934 75%), not Hispanic (835/934, 89%), and enrolled at a 4-year college (618/934, 66%). Over 95% (895/934) of respondents reported that they maintained access to their preferred contraception during the COVID-19 pandemic. In a multivariable analysis, participants who were enrolled in a 4-year college or graduate program were less likely to lose contraceptive access when compared to participants enrolled in a 2-year college (risk ratio [RR] 0.34, 95% CI 0.16-0.71); in addition, when compared to cisgender participants, nonbinary and transgender participants were more likely to lose contraceptive access (RR 2.43, 95% CI 1.01-5.87). Respondents reported that they were more interested in using telehealth to access contraception during the pandemic. The contraceptive methods most commonly used by our participants were, in order, condoms (331/934, 35.4%), oral contraception (303/934, 32.4%), and long-acting reversible contraception (LARC; 221/934, 23.7%). The rate of LARC use among our participants was higher than the national average for this age group (14%). Emergency contraception was uncommonly used (25/934, 2.7%).

**Conclusions:**

Undergraduate and graduate students in North Carolina overwhelmingly reported that they maintained access to their preferred contraceptive methods during the COVID-19 pandemic and through changing patterns of health care access, including telehealth. Gender nonbinary and transgender students and 2-year college students may have been at greater risk of losing access to contraception during the first year of the COVID-19 pandemic.

## Introduction

The COVID-19 pandemic caused by SARS-CoV-2 has brought about marked societal changes to control spread of infection. For young adults pursuing postsecondary education and potentially reliant on student health centers for health care services [1], cancellation of in-person classes and campus closures in the months of March to June 2020 caused dramatic changes in daily life.

National data suggest that contraceptive access decreased during the pandemic [2]. Women expressed worry about access to contraception and sexual and reproductive health care [3] and were more likely than men to defer care during the pandemic [4]. Lack of care was concentrated among racial minorities and those in poor health. Undergraduate and graduate students are a population that, due to their young age, tend to have few health care needs; however, they have high rates of sexual activity and unintended pregnancy and thus a need for contraception [5,6]. Contraceptive access and effective use across institutions of higher education and sociodemographic groups are not equal. Compared to 4-year college students, community college students are more likely to engage in risky sexual behavior and use emergency contraception, are less likely to use effective contraception, and experience higher rates of adverse sexual health outcomes [7,8]. Both Black and Hispanic students are less likely to use effective forms of contraception, and they experience higher rates of adverse sexual health outcomes, such as sexually transmitted infections (STIs) and unintended pregnancy [5,6,9-11]. Likewise, gender and sexual minorities (ie, nonheterosexual and noncisgender individuals) are less likely to use contraception at every sexual encounter; lack of awareness of this population’s reproductive health needs and the resultant inadequate reproductive health counseling targeted toward them puts this population at increased risk of adverse sexual health outcomes [12-15]. Given known racial and ethnic disparities in COVID-19 infection, morbidity, and mortality [16], we hypothesized that COVID-19 could also compound adverse effects on groups with less contraceptive access.

We sought to assess changes in contraceptive access and use among undergraduate and graduate students in North Carolina during the COVID-19 pandemic. We hypothesized that access to and use of preferred contraceptive methods would be decreased among students during COVID-19 because of difficulty accessing on-campus health clinics. We also hypothesized that we would observe disparities in contraceptive access and use by sociodemographic factors, including race, ethnicity, gender, sexual orientation, religion, and type of college attended.

## Methods

### Measures

We designed a cross-sectional, open online survey using Qualtrics (Qualtrics International, Inc). Methodology is reported below according to the Checklist for Reporting Results of Internet E-Surveys (CHERRIES) guidelines. The survey consisted of a total of 70 items addressing eligibility criteria (n=6 items), sexual behavior (n=43 items), and demographics (n=21 items), with 23 items only being displayed if specific display logic was met. We included the Brief Sensation Seeking Scale (BSSS), a measure of sensation seeking validated for use among young adults [17]. We also modified and included sections of the Johns Hopkins University COVID-19 Community Response Survey [18]. The full survey instrument is available for review in Multimedia Appendix 1.

### Recruitment

The survey was advertised to potential participants via targeted Instagram advertisements launched from an institutional Facebook account managed by the Duke Clinical and Translational Science Institute. The advertisements used for recruitment are available for review in Multimedia Appendix 2. Targeting sought the following parameters: location (North Carolina), gender (all), age (18-30 years), and interests (student life). The ad appeared in the Instagram feed of targeted users as a post with a link that directed users to our online survey.

At the Qualtrics landing page, potential participants first encountered and completed an electronic consent form. Those meeting inclusion criteria were invited to complete the entire survey. Participants had to be aged 18 to 30 years, enrolled in a 2- or 4-year undergraduate or graduate program in North Carolina, and speak English. Exclusion criteria included not finishing the survey, completing less than 50% of questions, completing the survey in less than 2 minutes, missing the primary outcome, or being identified as “fraudulent” by Qualtrics. Participants could be physically located anywhere, as long as they were currently enrolled in a 2- or 4-year undergraduate or graduate program in North Carolina.

The survey remained open until we reached our goal of 1000 eligible responses, which took 10 days total (March 4 through March 6, 2021, and March 23 through March 29, 2021).

### Ethical Considerations

Our study and advertising campaign were approved by the Duke University Health System Institutional Review Board (IRB) for human subject research (Pro00107332). The first page of the survey was an electronic consent form, which is available for review in Multimedia Appendix 1. The electronic consent form notified potential participants that some of the survey questions could make them feel uncomfortable, that they could refuse to answer any of the questions or stop their participation at any time, and that nonparticipation would not affect their academic standing. Participants were notified that “every effort [would] be made to keep [their] information confidential” and that names and emails collected for compensation would be stored independently from survey responses and would not be linked. We offered a $5 Amazon electronic gift card as an incentive for survey completion. Survey responses were deidentified and stored on Qualtrics.

### Preventing Multiple Submissions

Qualtrics has several tools to block bots from participation and prevent individuals from submitting multiple responses. In the first several days of recruitment (March 4-6), we determined that 3 people had requested payment for survey completion more than 1 time. We paused recruitment to update our IRB protocol and Qualtrics security measures to discourage repeat participation. We reopened the survey and collected the remaining responses. In this time frame (March 23-29), 2 people requested payment for survey completion more than 1 time.

### Sample Size

We anticipated a high rate of nonresponses given our recruitment methodology and inclusion and exclusion criteria. We planned our advertising campaign, considering our time frame and budget, to obtain at least 900 responses. We conducted power calculations in Power Analysis and Sample Size Software (NCSS, LLC) to obtain an effective sample size to detect a 10% reduction from the baseline proportion (70% of participants) of contraceptive use after social distancing interventions among participants. We used an inflation factor range to account for estimated response rates from 35% to 45%, which gave a range of inflation factors from 2.22 to 2.85. We did not have a prior estimate for ρ and so used a range from 0.1 to 0.3. A sample size between 700 to 800 was needed to have at least 80% power to detect such a change at α=.05. We oversampled to allow for potential subgroup analyses.

### Outcomes

Our primary outcome was the answer to the question “Compared to before social-distancing guidelines for COVID-19, please answer the following about your current behavior. I am not using the birth control method I prefer because of COVID-19.” Answer choices included strongly agree, agree, neutral, disagree, and strongly disagree. Both strongly agree and agree were coded as a change in access to preferred contraception, while neutral, disagree and strongly disagree were coded as no change in access. Secondary outcomes included use of emergency contraception in the previous month, prevalence of use of various types of contraception at the time of participants’ most recent sexual encounter, and if participants had ever used these types of contraception in their lifetime.

We identified several sociodemographic factors that, a priori, we hypothesized would be associated with contraceptive use based on prior data. These included attendance at a 2-year or community college, sexual orientation and gender minority status (ie, lesbian, gay, bisexual, transgender, queer [LGBTQ+]), lack of health insurance, non-White race, Hispanic ethnicity, higher scores on the BSSS, lower religiosity, and desire to become pregnant. We planned to stratify the analysis based on whether participants reported they had been sexually active in the last month. However, we did not observe the anticipated association between decreased contraceptive access and sexual activity and thus adjusted our analysis plan post hoc to not include this stratified analysis.

### Analysis

Statistical analysis was conducted using R (version 4.1.0; R Core Team) at a *P=*.05 2-tailed level of significance.

We conducted a bivariate analysis of each sociodemographic factor using modified Poisson regression models [19]. A multivariable model incorporating factors that were associated (*P*<.10) with decreased contraceptive access in the bivariate analysis was fitted. Risk ratios (RRs) and 95% CIs were reported.

## Results

### Survey Sample

**Figure 1:**
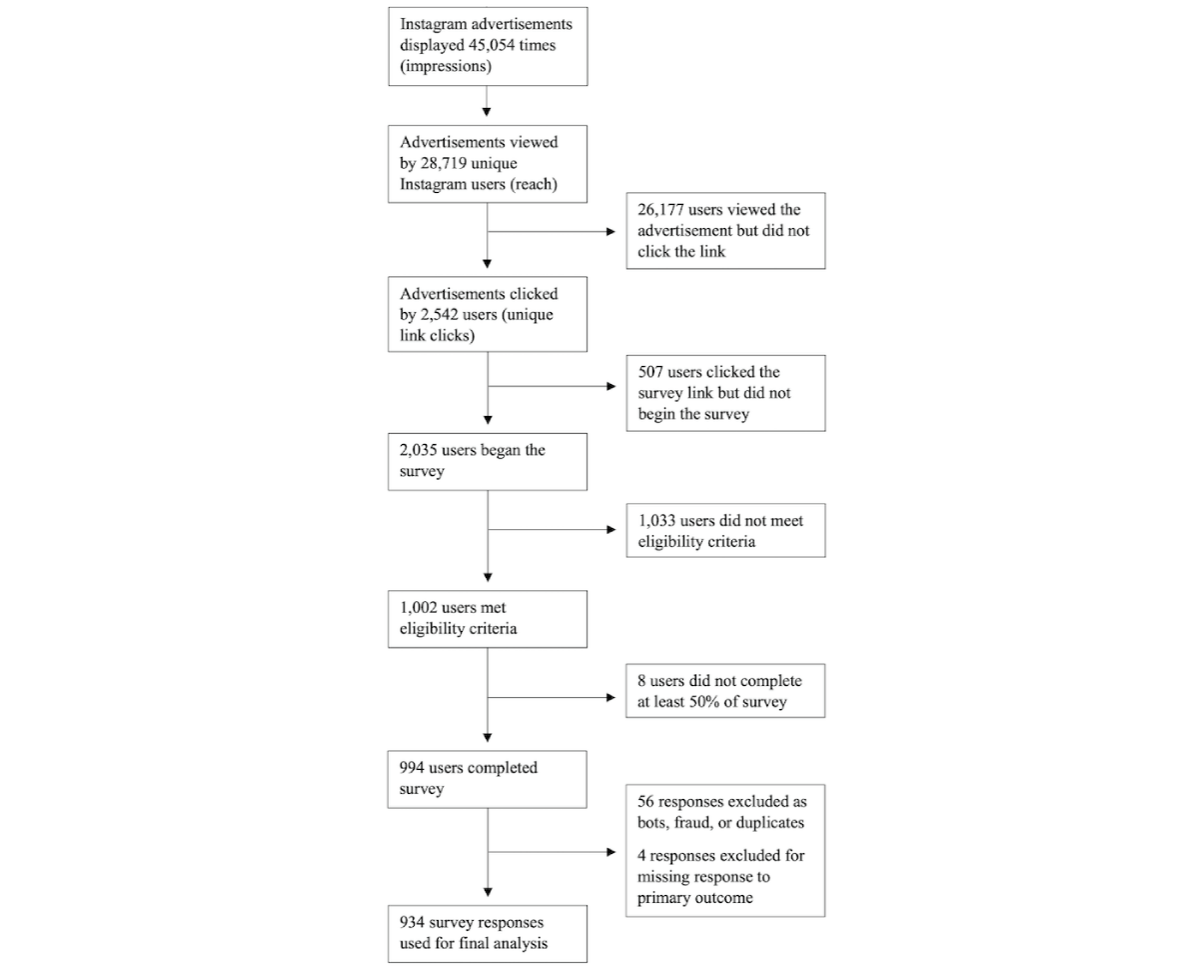
Flow sheet depicting our recruitment and enrollment.

Over 10 days, study Instagram advertisements were displayed 45,054 times (ie, impressions), viewed by 28,719 Instagram users (ie, reach), and clicked by 2542 users (ie, unique link clicks). A total of 2035 users began the survey (2035/2524, for an 81% participation/recruitment rate). A total of 1002 of 2035 respondents met the eligibility criteria. A total of 994 of 1002 eligible participants completed at least 50% of the survey. The exclusion of bots and duplicates led to 938 survey responses being included. We further excluded 4 participants for missing responses to the primary outcome, which left 934 responses for analysis, representing a 93% completion rate (Figure 1).

Respondents were mostly cisgender women (665/934, 71%), White (695/934, 75%), and enrolled at a 4-year college (618/934, 66%; Table 1). Our sample was ethnically diverse, with 11% (99/934) reporting Hispanic ethnicity, consistent with North Carolina state demographics [20]. We also had diversity of sexual orientation (592/934, 64% heterosexual; 167/934, 18% bisexual; 67/934, 7% gay/lesbian/homosexual) and gender identity (877/934, 94% cisgender; 35/934, 4% genderqueer/nonbinary; 11/934, 1.2% transgender).

**Table 1:** Demographic characteristics of North Carolina undergraduate and graduate student participants (n=934).

Characteristics		Values
**Age (years)**		
	Mean (SD)	21.7 (2.7)
	Range	18-30
**Gender identity, n (%)**		
	Female	665 (71.2)
	Genderqueer/nonbinary	35 (3.7)
	Intersex	1 (0.1)
	Male	212 (22.7)
	Transgender female	2 (0.2)
	Transgender male	9 (1)
	Other	3 (0.3)
	Prefer not to say	7 (0.7)
**Sexual orientation, n (%)**		
	Asexual	9 (1)
	Bisexual	167 (17.9)
	Gay/lesbian/homosexual	67 (7.2)
	Heterosexual or straight	592 (63.5)
	I don’t label myself	22 (2.4)
	Other	3 (0.3)
	Pansexual	20 (2.1)
	Queer	33 (3.5)
	Questioning	20 (2.1)
	Missing	1 (0.1)
**Race, n (%)**		
	White	695 (74.9)
	Black or African American	40 (4.3)
	Other	23 (2.5)
	Asian	123 (13.3)
	Multiracial	47 (5.1)
	Missing	6 (0.6)
**Ethnicity, n (%)**		
	Hispanic/Latino	99 (10.6)
**Type of school, n (%)**		
	Community college for GED (general education development)	11 (1.2)
	Four-year college	618 (66.4)
	Graduate program	269 (28.9)
	Two-year college	29 (3.1)
	Vocational school	4 (0.4)
	Missing	3 (0.3)
**Desire pregnancy, n (%)**		
	No	892 (97.4)
	Yes	11 (1.2)
	Unsure	13 (1.4)
	Missing	18 (1.9)
**Has insurance, n (%)**		
	Yes	895 (95.8)
	No	22 (2.4)
	Don’t know	16 (1.7)

### Contraceptive Access

Contrary to our primary hypothesis, the vast majority of participants reported that they maintained access to their preferred contraceptive method (895/934, 96%). In a multivariate analysis (Table 2), enrollment in a 4-year college or above was associated with greater access to the preferred contraceptive method when compared with enrollment in a 2-year college (adjusted RR [aRR] 0.34, 95% CI 0.16-0.71). Gender minorities reported increased risk of difficulty accessing preferred contraception (nonbinary gender or transgender identity aRR 2.43, 95% CI 1.01-5.87).

A minority of participants reported using emergency contraception at the last sexual encounter (25/934, 3%; Table 3), and 10% (88/921) of respondents reported that they were more likely to use emergency contraception during the pandemic. In a multivariate analysis, significant risk factors for using emergency contraception included self-identified Black race (aRR 3.53, 95% CI 1.24-10.03) and reporting religion to be very important (aRR 2.97, 95% CI 1.12-7.86).

When considering contraceptive method mix at both most recent sexual encounter and ever used, condoms were the most frequently used method (last encounter 331/934, 35%; ever used 700/933, 75%; Table 4). Dual-method use was also frequent, including concurrent condom and hormonal contraceptive use (last encounter 182/934, 20%; ever used 583/933, 63%) and withdrawal and hormonal contraceptive use (last encounter 127/934, 14%; ever used 388/933, 42%). Current use of long-acting reversible contraception (LARC) was reported by a proportion of the sample (last encounter 221/943, 24%; ever used 288/933, 31%). When considering how individual method mix might impact access to contraception, 1.7% (95% CI 0.4%-5.2%) of current intrauterine device (IUD) users reported decreased access to contraception, while higher proportions of contraceptive implant (9.3%, 95% CI 3%-21.3%) and injectable contraception (9.1%, 95% CI 0.5%-42.9%) users reported decreased access (Table 5).

**Table 2:** Risk factors for decreased access to preferred contraception (overall 39/934, 4.2%). Bivariate and multivariable associations were computed using modified Poisson regressions.

Risk factors			Values		Bivariate comparisons				Multivariable model^a^		
					RR^b^ (95% CI)		*P* value		Adjusted RR (95% CI)		*P* value
**Type of school of respondents (n=931), n (%)**											
	Two-year college	6 (13.6)		Reference				Reference			
	Four-year college	33 (3.7)		0.27 (0.12-0.62)		.002		0.34 (0.16-0.71)		.004	
**Gender of respondents (n=934), n (%)**											
	Cisgender male/female	32 (3.6)		Reference							
	Nonbinary or transgender	5 (10.6)		2.92 (1.19-7.14)		.02		2.43 (1.01-5.87)		.048	
	Other or prefer not to say	2 (20)		5.48 (1.52-19.82)		.01		4.25 (1.67-10.80)		.002	
**Sexual orientation of respondents (n=910), n (%)**											
	Lesbian, gay, bisexual, transgender, queer	13 (4)		Reference							
	Not lesbian, gay, bisexual, transgender, queer	24 (4.1)		1.02 (0.52-1.97)		.96		N/A^c^		N/A	
**Insurance status of respondents (n=933), n (%)**											
	No	0 (0)		N/A		N/A		N/A		N/A	
	Don’t know	0 (0)		N/A		N/A		N/A		N/A	
	Yes	39 (4.4)		N/A		N/A		N/A		N/A	
**Race of respondents (n=928), n (%)**											
	White	27 (3.9)		Reference							
	Black or African American	2 (5)		1.29 (0.32-5.22)		.72		N/A		N/A	
	Other	2 (8.7)		2.24 (0.57-8.85)		.25		N/A		N/A	
	Asian	5 (4.1)		1.05 (0.41-2.66)		.92		N/A		N/A	
	Multiracial	3 (6.4)		1.64 (0.52-5.22)		.40		N/A		N/A	
**Ethnicity of respondents (n=934), n (%)**											
	Non-Hispanic	32 (3.8)		Reference							
	Hispanic	7 (7.1)		1.85 (0.84-4.07)		.13		N/A		N/A	
Brief Sensation Seeking Scale score^d^ (n=934), mean (SD)			3.5 (0.8)		1.37 (0.86-2.19)		.18		N/A		N/A
**Importance of religion of respondents (n=931), n (%)**											
	Not important	24 (4.2)		Reference							
	Somewhat important	8 (3.5)		0.85 (0.39-1.86)		.68		N/A		N/A	
	Very important	7 (5.6)		1.34 (0.59-3.04)		.49		N/A		N/A	
**Desire for pregnancy of respondents (n=916), n (%)**											
	No	36 (4)		Reference							
	Yes	0 (0)		N/A		N/A		N/A		N/A	
	Unsure	2 (15.4)		3.81 (1.02-14.19)		.046		N/A		N/A	
**Sexual activity of respondents in the last month (n=923), n (%)**											
	No	10 (3.3)		Reference							
	At least with one partner	29 (4.6)		1.39 (0.69-2.81)		.36		N/A		N/A	

^a^The final multivariable model included type of school and gender identity.

^b^RR: risk ratio.

^c^N/A: not applicable.

^d^Cronbach α for the Brief Sensation Seeking Scale was α=.79 (95% CI .76-.81).

**Table 3:** Risk factors for emergency contraceptive use after most recent sexual encounter. Overall use of emergency contraception was 25/934 (2.7%). Bivariate and multivariable associations were computed using modified Poisson regressions.

Risk factors		Values	Bivariate comparisons		Multivariable model^a^	
			RR^b^ (95% CI)	*P* value	Adjusted RR (95% CI)	*P* value
**Type of school of respondents (n=931), n (%)**						
	Two-year college	0 (0)	N/A^c^	N/A	N/A	N/A
	Four-year college	25 (2.8)	N/A	N/A	N/A	N/A
**Gender of respondents (n=934), n (%)**						
	Cisgender male/female	23 (2.6)	Reference			
	Nonbinary or transgender	2 (4.3)	1.62 (0.39-6.68)	.50	N/A	N/A
	Other or prefer not to say	0 (0)	N/A	N/A	N/A	N/A
**Sexual orientation of respondents (n=910), n (%)**						
	Lesbian, gay, bisexual, transgender, queer	6 (1.9)	Reference			
	Not lesbian, gay, bisexual, transgender, queer	18 (3.1)	1.65 (0.66-4.12)	.28	N/A	N/A
**Insurance status of respondents (n=933), n (%)**						
	No	1 (4.5)	Reference		Reference	
	Don’t know	1 (6.2)	1.37 (0.09-20.38)	.82	N/A	N/A
	Yes	23 (2.6)	0.57 (0.08-4.00)	.57	N/A	N/A
**Race of respondents (n=928), n (%)**						
	White	16 (2.3)	Reference		Reference	
	Black or African American	4 (10)	4.34 (1.52-12.39)	.006	3.53 (1.24-10.03)	.02
	Other	2 (8.7)	3.78 (0.92-15.47)	.07	2.11 (0.54-8.25)	.29
	Asian	2 (1.6)	0.71 (0.16-3.03)	.64	0.82 (0.19-3.52)	.78
	Multiracial	1 (2.1)	0.92 (0.13-6.82)	.94	0.89 (0.12-6.60)	.91
**Ethnicity of respondents (n=934), n (%)**						
	Non-Hispanic	19 (2.3)	Reference		Reference	
	Hispanic	6 (6.1)	2.66 (1.09-6.51)	.03	2.11 (0.85-5.26)	.11
Brief Sensation Seeking Scale score^d^ (n=934), mean (SD)		3.4 (0.8)	1.28 (0.72-2.26)	.40	N/A	N/A
**Importance of religion of respondents (n=931), n (%)**						
	Not important	9 (1.6)	Reference		Reference	
	Somewhat important	9 (4)	2.55 (1.02-6.33)	.04	2.24 (0.93-5.40)	.07
	Very important	7 (5.6)	3.57 (1.35-9.40)	.01	2.97 (1.12-7.86)	.03
**Desire for pregnancy of respondents (n=916), n (%)**						
	No	23 (2.6)	Reference			
	Yes	0 (0)	N/A	N/A	N/A	N/A
	Unsure	1 (7.7)	2.98 (0.43-20.47)	.27	N/A	N/A
**Sexual activity of respondents in the last month (n=923), n (%)**						
	No	8 (2.7)	Reference			
	At least with one partner	16 (2.6)	0.96 (0.41-2.21)	.92	N/A	N/A

^a^The final multivariate model included race, Hispanic ethnicity, and importance of religion.

^b^RR: risk ratio.

^c^N/A: not applicable.

^b^Cronbach α for the Brief Sensation Seeking Scale was α=.79 (95% CI .76-.81).

**Table 4:** Use of contraception at last sexual encounter (n=934) and ever (n=933) among undergraduate and graduate student participants in North Carolina stratified by reported access to contraception during the pandemic.

Type of contraception	Used at last sexual encounter, n (%)				Ever used, n (%)		
	Unchanged access (n=895)	Decreased access (n=39)	Total (n=934)	Unchanged access (n=894)		Decreased access (n=39)	Total (n=933)
Condoms	312 (34.9)	19 (48.7)	331 (35.4)	667 (74.6)		33 (84.6)	700 (75)
Oral contraception	291 (32.5)	12 (30.8)	303 (32.4)	520 (58.2)		23 (59)	543 (58.2)
Injection	10 (1.1)	1 (2.6)	11 (1.2)	28 (3.1)		1 (2.6)	29 (3.1)
Implant	39 (4.4)	4 (10.3)	43 (4.6)	78 (8.7)		4 (10.3)	82 (8.8)
IUD^a^	175 (19.6)	3 (7.7)	178 (19.1)	227 (25.4)		8 (20.5)	235 (25.2)
Ring	19 (2.1)	0 (0)	19 (2)	41 (4.6)		1 (2.6)	42 (4.5)
Withdrawal	182 (20.3)	9 (23.1)	191 (20.4)	421 (47.1)		18 (46.2)	439 (47.1)
LARC^b^	214 (23.9)	7 (17.9)	221 (23.7)	276 (30.9)		12 (30.8)	288 (30.9)
Condoms and hormonal	174 (19.4)	8 (20.5)	182 (19.5)	559 (62.5)		24 (61.5)	583 (62.5)
Withdrawal and hormonal	125 (14)	2 (5.1)	127 (13.6)	375 (41.9)		13 (33.3)	388 (41.6)
Emergency contraception	23 (2.6)	2 (5.1)	25 (2.7)	217 (24.3)		9 (23.1)	226 (24.2)
Natural family planning	17 (1.9)	2 (5.1)	19 (2)	68 (7.6)		5 (12.8)	73 (7.8)
Trying to get pregnant	7 (0.8)	0 (0)	7 (0.7)	0 (0)		0 (0)	0 (0)
Never had sex	179 (20)	2 (5.1)	181 (19.4)	151 (16.9)		3 (7.7)	154 (16.5)
Surgical sterilization	2 (0.2)	0 (0)	2 (0.2)	5 (0.6)		0 (0)	5 (0.5)
Other	6 (0.7)	0 (0)	6 (0.6)	8 (0.9)		0 (0)	8 (0.9)

^a^IUD: intrauterine device.

^b^LARC: long-acting reversible contraception.

**Table 5:** Variation in decreased contraceptive access by contraceptive type.

Type of contraception	Unchanged access, n	Decreased access, n	Decreased access, % (95% CI)
Condom	312	19	5.7 (3.6-9)
Implant	39	4	9.3 (3-21.3)
Injection	10	1	9.1 (0.5-42.9)
IUD^a^	175	3	1.7 (0.4-5.2)
LARC^b^	214	7	3.2 (1.4-6.7)
Oral contraception	291	12	4 (2.2-7)
Ring	19	0	0 (0-20.9)

^a^IUD: intrauterine device.

^b^LARC: long-acting reversible contraception.

### Telehealth

**Figure 2:**
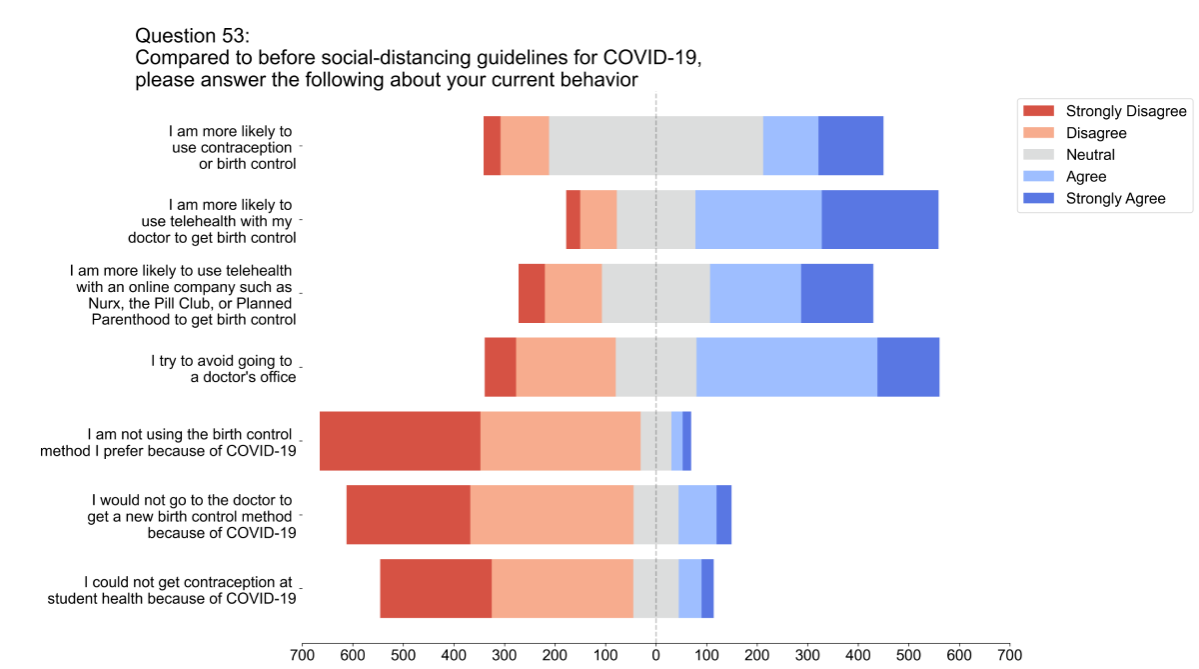
Participant behavior before and after the COVID-19 pandemic. Participants were asked to indicate agreement or disagreement with a variety of statements regarding their behavior both before and after the onset of social-distancing guidelines for the COVID-19 pandemic.

Respondents indicated a trend toward using telehealth for contraceptive access (Figure 2), with 64% (481/737) reporting that since the start of the pandemic, they were more likely to use telehealth with their doctor to access contraception and 46% (323/702) reporting that they were more likely to use a telehealth company to access contraception. In addition, 54% (481/900) of respondents reported that they had tried avoiding the doctor’s office since the start of the pandemic. However, 74% (568/762) of respondents disagreed with the statement “I would not go to the doctor to get a new birth control method because of COVID-19.”

## Discussion

### Principal Results

Contrary to our hypothesis, we found that over 95% (895/934) of surveyed undergraduate and graduate student participants maintained access to their preferred contraceptive method during the COVID-19 pandemic. Both increased interest in telehealth and high rates of LARC use among respondents may have been key factors for continued contraceptive access. Our findings suggest that health systems serving young adults in higher education have been able to pivot to maintain access to sexual and reproductive health services.

Respondents reported that they were more likely to use telehealth to access contraception, including both telehealth with their established doctors and straight-to-consumer “tele-contraception” companies. However, our survey was not worded to assess actual rate of use, so telehealth was not used as a predictor in bivariate or multivariate models, and we are unable to draw definite conclusions about whether participants did use telehealth at a higher rate. Expanded avenues for access without an in-person visit are consistent with guidelines from the American College of Obstetricians and Gynecologists and American Academy of Family Physicians [21-23]. Telehealth for contraceptive counseling was also acceptable to providers during the COVID-19 pandemic [24].

Another possible explanation for the reported prevalence of contraceptive access was the high rate of LARC use among our respondents (221/943, 24%). Our observed percentage of LARC use was higher than the 14% reported among women aged 20 to 29 years from the 2017-2019 National Survey of Family Growth [25]. LARC use is positively associated with higher levels of education, which may help explain this finding given our sample [6,25]. Interestingly, LARC users did not have uniformly low levels of reported decreased access. A relatively high proportion of contraceptive implant users (4/43, 9%) reported decreased access (Table 5). These results must be interpreted with caution given the low prevalence of use, but they are consistent with results for Depo-Provera (9% of users reported decreased access), another method in which users may experience unscheduled bleeding. Future research might examine how issues like unscheduled bleeding, other side effects, or desire to change methods contribute to lack of access to desired care.

Another factor that likely contributed to contraceptive access among our participant population was the high rate of medical insurance (895/934, 95.8%). This is higher than both the national (84.4%) and North Carolina (80.3%) rates of insurance among young adults aged 19 to 34 years [26]; however, it is representative of the national rate of insurance among US college students, which was greater than 90% in 2018 [27].

Nonbinary gender or transgender identity and enrollment at a 2-year college were sociodemographic risk factors for decreased contraceptive access. Though gender-affirming hormone therapy does not necessarily prevent pregnancy [28,29], providers often do not provide contraceptive counseling for gender nonbinary and transgender patients [14,15]. Our data showed that the COVID-19 pandemic may have amplified difficulty with contraceptive access for this at-risk group. Though we had few 2-year college participants, our findings are consistent with known disparities in sexual and reproductive health for community college students, who are more likely to experience unintended pregnancy and engage in risky sexual behavior and less likely to use more effective forms of contraception, such as LARC [7,8]. Common barriers to more effective use of contraceptive methods in this population include lack of insurance, inability to afford the desired method, lack of knowledge of where to obtain the desired method, and a low number of student health centers on 2-year college campuses [7]. These barriers were likely exacerbated by the pandemic.

We examined use of emergency contraception, as it might indicate decreased access to more effective contraceptive methods. Our results suggesting that Black students were more likely to use emergency contraception are in contrast with prior research showing that non-Hispanic Black women are less likely than Hispanic or non-Hispanic White women to use emergency contraception [30]. Likewise, more frequent emergency contraceptive use among those who highly value religion may warrant further investigation. College campuses with a religious affiliation are less likely to have health centers that prescribe contraception and individual physicians who strongly value religion are also less likely to prescribe contraception [1,31]. While contraceptive and emergency contraception use may be counter to anticontraception beliefs advanced by religious groups [32,33], our findings of frequent emergency contraceptive use among undergraduate and graduate students with religious affiliations suggest that this population still desires contraceptive access and thus has contraceptive needs that are not met by their educational institutions.

### Limitations

This study has several limitations. This was a cross-sectional survey performed in March 2021. As a result, we did not accurately capture participants’ behavior when their lives were most disrupted by campus closures [34]. Respondents self-selected participation in a survey they knew to be about sexual behavior, which may limit generalizability. Our participant population was skewed toward White females with very high rates of being insured. This skew is reflective of the unequal sex distribution in North Carolina college enrollment and the demographics at the 3 largest institutions represented in our sample (Duke University, University of North Carolina Chapel Hill, and North Carolina State University), which have more White students than the statewide average [35,36]. Given the low number of respondents reporting lack of access to contraception, we were not adequately powered to identify health disparities.

### Conclusions

The majority of respondents maintained access to their preferred contraceptive method during the pandemic. Risk factors for decreased access included nonbinary gender or transgender identity versus cisgender identity and enrollment at a 2-year college versus 4-year college and above, suggesting that the pandemic has exacerbated some preexisting disparities in contraceptive access. Respondents reported increased use of telehealth for contraception, which will be an important tool for maintaining and promoting equitable contraceptive access through the remainder of the COVID-19 pandemic and beyond.

## Appendix

Multimedia Appendix 1 Full online survey instrument.

Multimedia Appendix 2 Study Instagram Advertisements. These are the advertisements used to recruit participants for our study, as they appeared on the app interface.

## Abbreviations

aRR: adjusted risk ratio
BSSS: Brief Sensation Seeking Scale
IRB: institutional review board
IUD: intrauterine device
LARC: long-acting reversible contraception
LGBTQ+: lesbian, gay, bisexual, transgender, queer
RR: risk ratio
STI: sexually transmitted infection

## Data Availability

Our data are not publicly available because our approved institutional review board protocol did not allow for public data sharing.
