# Supplementary material for: Contraceptive Access and Use Among Undergraduate and Graduate Students During COVID-19: Online Survey Study
Source: JMIR Form Res. 2023 Mar 14;7:e38491. doi: 10.2196/38491 (PMC10018798; doi:10.2196/38491)
Supplement: Multimedia Appendix 1 [file formative_v7i1e38491_app1.docx]

Multimedia Appendix 1: Full online survey instrument

Sex and Contraception During COVID-19

Start of Block: Consent

Q1 You are being asked to complete a brief survey designed to better understand changes in sexual behavior and sexual health access among undergraduate and graduate students in North Carolina before and during the COVID-19 pandemic. The survey should take less than 10 minutes to complete and is completely voluntary.  Some of the questions we will ask you as part of this study may make you feel uncomfortable. You may refuse to answer any of the questions and you may take a break at any time during the study. You may choose not to be in the study, or if you agree to be in the study, you may exit the survey and stop your participation at any time. Nonparticipation or withdrawal from this study will not affect your grades if you are a Duke student.  There are no physical risks associated with this study. There is, however, the potential risk of loss of confidentiality. Every effort will be made to keep your information confidential; however, this cannot be guaranteed. If you complete our survey, you will receive a $5 Amazon electronic gift card for your time. You must complete at least 80% of survey questions to be eligible to receive a gift card. Your name and email will be collected at the end of the survey in order to distribute the gift card. This information will be collected and stored independently from your survey responses. They will not be linked. You may also opt in to be contacted for potential future studies. This is not required. Gift cards will be distributed within five business days of survey completion. This is a one-time survey. If you start the survey and then decide to withdraw from the study, we will not be able to remove the responses you already made because survey responses are not linked to identifiable data.  For questions about the study, or if you have concerns, questions or suggestions about the research, contact our research team at covidsexsurvey@duke.edu. For questions about your rights as a research participant, or to discuss problems, concerns or suggestions related to the research, or to obtain information or offer input about the research, contact the Duke University Health System Institutional Review Board (IRB) Office at (919) 668-5111. By clicking to the next section below, you are indicating your consent to join this research study. You will be taken directly to the survey and the first few questions will determine your eligibility. Please answer each question carefully.

- YES, I have read the provided information and I wish to participate in this study (1)
- NO, I DO NOT wish to participate in this study (2)

End of Block: Consent

Start of Block: Eligibility Criteria

| 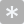 |
| --- |

Q2 How old are you?

________________________________________________________________

Q3 Are you currently enrolled in a two- or four-year college or graduate program in North Carolina?

- Yes (1)
- No (2)

Display This Question:

If Are you currently enrolled in a two- or four-year college or graduate program in North Carolina? = Yes

Q4 What kind of school are you currently enrolled in?

- Community college for GED (1)
- Two year college (2)
- Four year college (3)
- Vocational school (4)
- Graduate program (5)

Display This Question:

If Are you currently enrolled in a two- or four-year college or graduate program in North Carolina? = Yes

Q5 What post-secondary educational institution are you currently attending?


Choose one. If there are multiple, choose the one from which you are taking a majority of credits.

▼ Alamance Community College (1) ... Not listed (please specify) (174)

Display This Question:

If What post-secondary educational institution are you currently attending? Choose one. If there are... = Not listed (please specify)

Q80 Please specify which post-secondary education institution you are currently attending.

________________________________________________________________

Q79 Do you speak English?

- Yes (1)
- No (2)

End of Block: Eligibility Criteria

Start of Block: Sexual Behavior

Q28
The following questions ask about your personal attitudes and preferences. You can answer according to your pre-COVID-19 preferences.

|  | 1 - Strongly Disagree (1) | 2 - Moderate Disagree (2) | 3 - Neutral (3) | 4 - Moderately Agree (4) | 5 - Strongly Agree (5) |
| --- | --- | --- | --- | --- | --- |
| I would like to explore strange places (1) |  |  |  |  |  |
| I would like to take off on a trip with no pre-planned routes or timetables (2) |  |  |  |  |  |
| I get restless when I spend too much time at home (3) |  |  |  |  |  |
| I prefer friends who are excitingly unpredictable (4) |  |  |  |  |  |
| I like to do frightening things (5) |  |  |  |  |  |
| I would like to try bungee jumping (6) |  |  |  |  |  |
| I like wild parties (7) |  |  |  |  |  |
| I would love to have new and exciting experiences, even if they are illegal (8) |  |  |  |  |  |

| Page Break |  |
| --- | --- |

Q29 How often do you experience sexual desire?

- Never (1)
- Less than once a month (2)
- About once a month (3)
- About once a week (4)
- Several times a week (5)
- Daily (6)
- Several times a day (7)

Q30 How often do you orgasm in the average month?

- Never (1)
- 1-2 times (2)
- About once per week (3)
- Several times a week (4)
- Daily (5)
- Several times a day (6)

Q32 How many times do you masturbate in the average month?

- Never (1)
- 1-2 times (2)
- About once per week (3)
- Several times a week (4)
- Daily (5)
- Several times a day (6)

Q33 How would you compare your level of sex drive with that of the average person of your gender and age?

- Very much lower (1)
- A little lower (2)
- Somewhat lower (3)
- About the same (4)
- Somewhat greater (5)
- A little greater (6)
- Very much greater (7)

| Page Break |  |
| --- | --- |

Q34 Now we are going to ask you some questions about your past sexual activity.
In the last 12 months, how often did you:

|  | Never (1) | Less than half the time (2) | About half the time (3) | More than half the time (4) | Every time (5) | N/A - I do not perform this act (6) | N/A - I have not been sexually active in the last year (7) | N/A - I have never been sexually active (8) |
| --- | --- | --- | --- | --- | --- | --- | --- | --- |
| Use a condom during fellatio (mouth to penis, blowjobs)? (1) |  |  |  |  |  |  |  |  |
| Use a glove, dam, or other barrier during cunnilingus (mouth to vulva or vagina, going down)? (2) |  |  |  |  |  |  |  |  |
| Use a condom with a regular sexual partner during penetrative sex? (3) |  |  |  |  |  |  |  |  |
| Use a condom with a casual sexual partner during penetrative sex? (4) |  |  |  |  |  |  |  |  |
| Use a condom with a new sexual partner during penetrative sex? (5) |  |  |  |  |  |  |  |  |

Q35 Comparing this month to the same month one year ago, are you more likely, the same or less likely to have done the following?

|  | Less likely (1) | The same (2) | More likely (3) | N/A (4) |
| --- | --- | --- | --- | --- |
| Gone on an in-person date? (1) |  |  |  |  |
| Met someone at a party/bar who you hooked up with the same day? (2) |  |  |  |  |
| Kissed a new romantic partner for the first time? (3) |  |  |  |  |
| Kissed and had other sexual contact on a first date? (4) |  |  |  |  |
| Had sexual contact without kissing? (5) |  |  |  |  |
| Touched a partner's genitals with your hands or had a partner touch your genitals with their hands? (6) |  |  |  |  |
| Had oral sex with a partner? (7) |  |  |  |  |
| Had intercourse (vaginal or anal penetration by a human penis)? (8) |  |  |  |  |
| Been in a monogamous sexual relationship? (9) |  |  |  |  |
| Had multiple sexual partners in one month? (10) |  |  |  |  |
| Had sex with multiple partners at the same time? (11) |  |  |  |  |

Q36 Comparing this month to the same month one year ago, are you more likely, the same or less likely to have used emergency contraception or the morning after pill?

- Less likely (1)
- The same (2)
- More likely (3)
- N/A (4)

Q37 Comparing this month to the same month one year ago, are you more likely, the same or less likely to have done the following?

|  | Less likely (1) | The same (2) | More likely (3) | N/A (4) |
| --- | --- | --- | --- | --- |
| Used a digital sex or dating app (e.g. Bumble, Tinder, Hinge, Grindr, etc.)? (1) |  |  |  |  |
| Connected/matched with someone on a sex or dating app? (2) |  |  |  |  |
| Gone on a video date? (3) |  |  |  |  |
| Met someone in person who you matched/messaged with on an app? (4) |  |  |  |  |
| Met someone in person after having had a video date? (5) |  |  |  |  |
| Had phone or video sex? (6) |  |  |  |  |
| Sent explicit or sexual images of yourself to someone? (7) |  |  |  |  |
| Viewed pornography? (8) |  |  |  |  |
| Masturbated? (9) |  |  |  |  |

| Page Break |  |
| --- | --- |

Q38 The following questions will ask you about your body parts to understand your health andsexual history.

Q39 Which of the following body parts do you have? (Choose all that apply)

- Penis (1)
- Uterus (womb) (2)
- None of the above (3)

Display This Question:

If Which of the following body parts do you have? (Choose all that apply) = Uterus (womb)

Q40 Have you ever been pregnant?

- Yes (1)
- No (2)
- Prefer not to say (3)

Display This Question:

If Which of the following body parts do you have? (Choose all that apply) = Uterus (womb)

Q41 Have you ever had an abortion?

- Yes (1)
- No (2)
- Prefer not to say (3)

Display This Question:

If Which of the following body parts do you have? (Choose all that apply) = Penis

Q42 Have you ever gotten someone pregnant?

- Yes (1)
- No (2)
- Prefer not to say (3)

Display This Question:

If Which of the following body parts do you have? (Choose all that apply) = Penis

Q43 Has your partner ever had an abortion that you know of from sex with you?

- Yes (1)
- No (2)
- Prefer not to say (3)

Display This Question:

If Now we are going to ask you some questions about your past sexual activity. In the last 12 months... = N/A - I have never been sexually active

Q44 As you know, some people have had sex by your age and others have not. What would you say is the most important reason you have not had sexual intercourse up to now?

- Against religion or morals (1)
- Don't want to get pregnant (2)
- Don't want to get a sexually transmitted disease (3)
- Haven't found the right person yet (4)
- In a relationship but waiting for the right time (5)
- Concerned about exposure to COVID-19 (6)
- Other (please specify) (7)

Display This Question:

If As you know, some people have had sex by your age and others have not. What would you say is the... = Other (please specify)

Q45 Please specify the most important reason you have not had sexual intercourse

________________________________________________________________

| Page Break |  |
| --- | --- |

Q46 The **last time** you had vaginal intercourse (penis in vagina), which, if any, form(s) of contraception did your or your partner use? You may select more than one answer.

- N/A. I have never had vaginal intercourse (1)
- One of us was pregnant or trying to become pregnant so we did not use birth control (2)
- Condom (3)
- Oral contraception (the pill) (4)
- Injection (Depo Provera) (5)
- Implant (Nexplanon) (6)
- Intrauterine device (IUD such as Paragard, Mirena, Liletta, Skyla, Kyleena) (7)
- Diaphragm (8)
- Hormonal ring (9)
- Emergency contraception (morning after pill) (10)
- Withdrawal (pulling out) (11)
- Natural family planning (cycle tracking or the rhythm method) (12)
- Surgical sterilization (tubal ligation or vasectomy) (13)
- Other (please specify) (14)
- None of these (15)

Display This Question:

If The last time you had vaginal intercourse (penis in vagina), which, if any, form(s) of contracept... = Other (please specify)

Q47 Please specify the form(s) of contraception you or your partner used the last time you had vaginal intercourse

________________________________________________________________

Q50 Which, if any, methods of birth control have you or your partner **ever** used? Please select the method even if you have only used it once.

- N/A. I have never had vaginal intercourse (1)
- Condom (3)
- Oral contraception (the pill) (4)
- Injection (Depo Provera) (5)
- Implant (Nexplanon) (6)
- Intrauterine device (IUD such as Paragard, Mirena, Liletta, Skyla, Kyleena) (7)
- Diaphragm (8)
- Hormonal ring (9)
- Emergency contraception (morning after pill) (10)
- Withdrawal (pulling out) (11)
- Natural family planning (cycle tracking or the rhythm method) (12)
- Surgical sterilization (tubal ligation or vasectomy) (13)
- Other (please specify) (14)
- I have never used contraception (15)

Display This Question:

If Which, if any, methods of birth control have you or your partner ever used? Please select the met... = Other (please specify)

Q51 Please specify any additional forms of contraception used by you or your partner.

________________________________________________________________

Q52 Would you like to become pregnant in the next year?

- Yes (1)
- No (2)
- OK either way (3)
- Unsure (4)

| Page Break |  |
| --- | --- |

Q53 Compared to before social-distancing guidelines for COVID-19, please answer the following about your current behavior

|  | Strongly Agree (1) | Agree (2) | Neutral (3) | Disagree (4) | Strongly Disagree (5) | N/A (6) |
| --- | --- | --- | --- | --- | --- | --- |
| I am more likely to use contraception or birth control (1) |  |  |  |  |  |  |
| I am more likely to use telehealth with my doctor to get birth control (2) |  |  |  |  |  |  |
| I am more likely to use telehealth with an online company such as Nurx, the Pill Club, or Planned Parenthood to get birth control (3) |  |  |  |  |  |  |
| I try to avoid going to a doctor's office (4) |  |  |  |  |  |  |
| I am not using the birth control method I prefer because of COVID-19 (5) |  |  |  |  |  |  |
| I would not go to the doctor to get a new birth control method because of COVID-19 (6) |  |  |  |  |  |  |
| I could not get contraception at student health because of COVID-19 (7) |  |  |  |  |  |  |

| Page Break |  |
| --- | --- |

Q54 Have you **ever** been diagnosed with an STI? You may select more than one answer.

- No (1)
- Genital warts (2)
- HPV (3)
- Herpes (4)
- Chlamydia (5)
- Gonorrhea (6)
- Pelvic inflammatory disease (7)
- Syphilis (8)
- Trichomoniasis (9)
- HIV (10)
- Other (please specify) (11)

Skip To: Q58 If Have you ever been diagnosed with an STI? You may select more than one answer. = No

Display This Question:

If Have you ever been diagnosed with an STI? You may select more than one answer. = Other (please specify)

Q55 Please specify what STI(s) you have been diagnosed with

________________________________________________________________

Q56 Since the beginning of social distancing/quarantine with COVID-19, have you been diagnosed with an STI? You may select more than one answer.

- No (1)
- Genital warts (2)
- HPV (3)
- Herpes (4)
- Chlamydia (5)
- Gonorrhea (6)
- Pelvic inflammatory disease (7)
- Syphilis (8)
- Trichomoniasis (9)
- HIV (10)
- Other (please specify) (11)

Display This Question:

If Since the beginning of social distancing/quarantine with COVID-19, have you been diagnosed with a... = Other (please specify)

Q57 Please specify what STI(s) you have been diagnosed with since the start of the COVID-19 pandemic

________________________________________________________________

| Page Break |  |
| --- | --- |

Q58 In general, how would you describe your health?

- Poor (1)
- Fair (2)
- Good (3)
- Very good (4)

Q61 In the last three days, has any member of your household left your home and gone to another location for their job or school?

- Yes (1)
- No (2)
- Unsure (3)

Q62 In the last week, how many times did you spend time in a group of more than 20 people?

- 0-5 (1)
- >5 (2)

Q63 Have you been within 6 feet of any of the following people today? You may select more than one answer.

- Individuals with whom you live (eg family, roommates, friends, significant others) (1)
- Family (non-household) (2)
- Friends (non-household) (3)
- Romantic and/or sexual partners (non-household) (4)
- Co-workers or classmates (5)
- Patients or patrons (6)
- Any other type of person not already mentioned (7)
- None of the above (8)

| 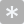 |
| --- |

Q64 Approximately how many times, if at all, did you wash your hands in the past 24 hours? Your best guess is fine.

________________________________________________________________

Q65 Are you currently practicing social distancing, or in other words, are you deliberately increasing the physical space between you and other people from outside your household to avoid spreading illness?

- Yes (1)
- Sometimes (2)
- No (3)

Q67 Have you had COVID-19 and/or been vaccinated against COVID-19?

- I've had COVID-19 (1)
- I've been vaccinated against COVID-19 (2)
- Both (3)
- Neither (4)

Q68 Do you think it's likely that you'll get the coronavirus, also known as COVID-19?

- Yes (1)
- No (2)
- Unsure (3)

Q69 How would you describe the way most people at your college or university are dealing with the coronavirus outbreak?

- Most are underestimating the risks (1)
- Most are reacting appropriately (2)
- Most are overreacting to the actual risks (3)

Q70 Have you made any changes to your routine as a result of the coronavirus outbreak?

- Yes, I've made large changes (1)
- Yes, I've made small changes (2)
- No, I have not made real changes (3)

| Page Break |  |
| --- | --- |

Q71 How often have you been tested for COVID-19?

- Never (1)
- Once (2)
- 1-3 times (3)
- Monthly (4)
- Weekly (5)
- More than once a week (6)

Q72 If you have tested positive for COVID-19, did your positive test results change the way you deal with the coronavirus?

- Yes, they make me **less** likely to socially distance (1)
- Yes, they make me **more** likely to socially distance (2)
- No, they do not change my behavior (3)
- N/A, I haven't tested positive (4)

Q73 If you have tested negative for COVID-19, did your negative test results change the way you deal with the coronavirus?

- Yes, they make me **less** likely to socially distance (1)
- Yes, they make me **more** likely to socially distance (2)
- No, they do not change my behavior (3)
- N/A, I haven't tested positive (4)

Q74 Do positive test results of others change the way you deal with the coronavirus?

- Yes, they make me **less** likely to socially distance (1)
- Yes, they make me **more** likely to socially distance (2)
- No, they do not change my behavior (3)
- N/A, the people around me do not get tested (4)

Q75 Do negative test results of others change the way you deal with the coronavirus?

- Yes, they make me **less** likely to socially distance (1)
- Yes, they make me **more** likely to socially distance (2)
- No, they do not change my behavior (3)
- N/A, the people around me do not get tested (4)

| Page Break |  |
| --- | --- |

Q76 In the past 30 days, have you done the following?

|  | Yes (1) | Sometimes (2) | No (3) | Not sure (4) | N/A (5) |
| --- | --- | --- | --- | --- | --- |
| Worn a mask on a date? (1) |  |  |  |  |  |
| Attended an in-person bar or party gathering? (2) |  |  |  |  |  |
| Worn a mask at an in-person bar or party gathering? (3) |  |  |  |  |  |
| Asked a sexual partner about the date of their most recent COVID test? (4) |  |  |  |  |  |
| Asked a sexual partner about symptoms for COVID? (5) |  |  |  |  |  |
| Declined kissing with a **previous** sexual partner(s) because of concerns about COVID? (6) |  |  |  |  |  |
| Declined kissing with a **new** sexual partner(s) because of concerns about COVID? (7) |  |  |  |  |  |
| Declined sex with a **previous** sexual partner(s) because of concerns about COVID? (8) |  |  |  |  |  |
| Declined sex with a **new** sexual partner(s) because of concerns about COVID? (9) |  |  |  |  |  |

End of Block: Sexual Behavior

Start of Block: Demographics

Q8 What is your current gender identity?

▼ Female (1) ... Prefer not to say (8)

Display This Question:

If What is your current gender identity? = Other (please specify)

Q9 Please specify your current gender identity

________________________________________________________________

Q10 To which sexual orientation do you currently identify yourself?

▼ Heterosexual or straight (1) ... Other (please specify) (9)

Display This Question:

If To which sexual orientation do you currently identify yourself? = Other (please specify)

Q11 Please specify your current sexual orientation

________________________________________________________________

Q12 What is your current relationship status?

- Single (1)
- Married (2)
- Engaged (3)
- Divorced (4)
- Widowed (5)
- In an exclusive relationship with one partner (6)
- In an open relationship (7)

Q83 How many sexual partners have you had in the last month?

- None (1)
- One (2)
- Multiple (3)
- Prefer not to say (4)

Q13 Who do you live with?

- Alone in a dorm (1)
- Alone not on campus (2)
- With roommates in a dorm (3)
- With roommates not on campus (4)
- With my romantic partner(s) (5)
- With my children and with or without my romantic partner(s) (6)
- With other family members who are not students (mother, father, siblings, etc.) (7)

| 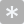 |
| --- |

Q14 How many people (not including yourself) do you live with?

________________________________________________________________

Q15 What is your political affiliation?

▼ Democratic (1) ... Other (please specify) (6)

Display This Question:

If What is your political affiliation? = Other (please specify)

Q16 Please specify your political affiliation

________________________________________________________________

Q17 Are you of Hispanic ethnicity, LatinX or Spanish origin?

- Yes (1)
- No (2)

Q18 What is your race? One or more races may be selected.

- White (1)
- Black or African American (2)
- American Indian or Alaska Native (3)
- Asian (4)
- Native Hawaiian or Pacific Islander (5)
- Other (please specify) (6)

Display This Question:

If What is your race? One or more races may be selected. = Other (please specify)

Q82 Please specify your race

________________________________________________________________

Q19 What is your present religion, if any?

▼ Agnostic (10) ... Other (please specify) (11)

Display This Question:

If What is your present religion, if any? = Other (please specify)

Q20 Please specify your religion

________________________________________________________________

Q21 Currently, how important is religion in your daily life?

- Very important (1)
- Somewhat important (2)
- Not important (3)

Q22 Are you currently covered by any type of health insurance or some other kind of health care plan?

- Yes (1)
- No (2)
- I don't know (3)

Display This Question:

If Are you currently covered by any type of health insurance or some other kind of health care plan? = Yes

Q23 What type of health insurance do you hold? (Choose all that apply)

- Private (1)
- Medicaid (2)
- Children's Health Insurance Program (CHIP) (3)
- Military (Tricare, VA, CHAMP-VA) (your own or a family member's) (4)
- Employer insurance (your own or a family member's) (5)
- Student insurance through your school (6)
- Other government programs (7)
- Other state-sponsored health plans (8)

Display This Question:

If Are you currently covered by any type of health insurance or some other kind of health care plan? = No

Q24 What is your reason for having no health insurance?

- I can no longer afford insurance due to COVID (1)
- I could not afford insurance before COVID and currently cannot (2)
- I am no longer at my job that supplies my coverage (3)
- I missed a deadline for receiving insurance (4)
- I am ineligible due to my age (5)
- I am ineligible due to leaving/not being on campus (6)
- I decided I do not need health insurance (7)
- Other (please specify) (8)

Display This Question:

If What is your reason for having no health insurance? = Other (please specify)

Q25 Please specify your reason for having no health insurance

________________________________________________________________

Q26 In the past 6 months, were you without health insurance coverage for a month or more?

- Yes (1)
- No (2)
- I don't know (3)

End of Block: Demographics
