# Supplementary figures and images for: Contraceptive Access and Use Among Undergraduate and Graduate Students During COVID-19: Online Survey Study
Source: JMIR Form Res. 2023 Mar 14;7:e38491. doi: 10.2196/38491 (PMC10018798; doi:10.2196/38491)

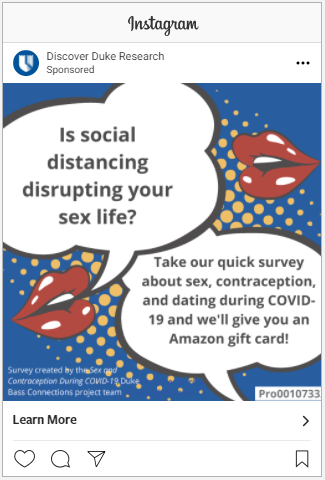

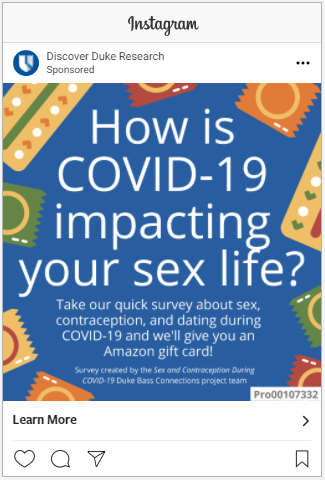

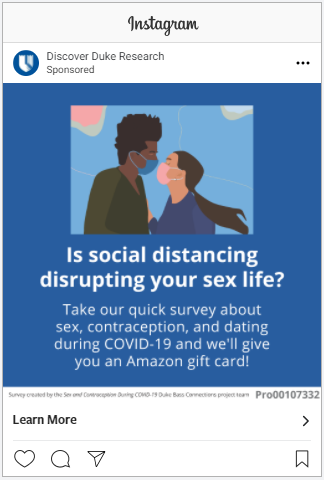

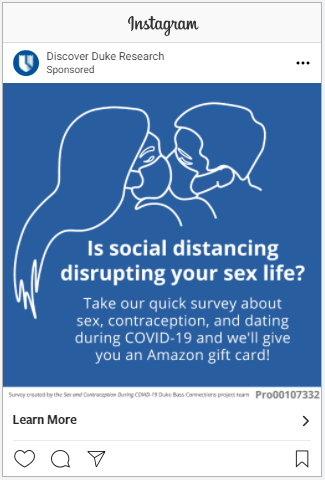

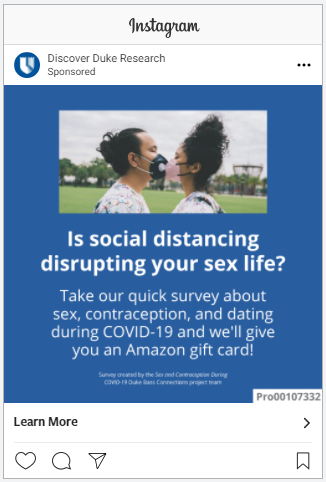

Supplement: Multimedia Appendix 2 [file formative_v7i1e38491_app2.docx]
